# Therapeutic apheresis within immune-mediated neurological disorders: dosing and its effectiveness

**DOI:** 10.1038/s41598-020-64744-4

**Published:** 2020-05-13

**Authors:** Matthias Klingele, Carina Allmendinger, Solmaz Thieme, Lea Baerens, Danilo Fliser, Bürmann Jan

**Affiliations:** 1 Saarland University Medical Centre, Homburg/Saar, Germany; 2 Department of Internal Medicine, Nephrology and Hypertension, Homburg, Germany; 3 Department of Neurology, Homburg, Germany; 4 Hochtaunus-Kliniken, Department of Nephrology, Bad Homburg, Germany

**Keywords:** Neurology, Neurological disorders

## Abstract

Plasma exchange (PE) and immunoadsorption (IA) are standard therapeutic options of immune-mediated neurological disorders. This study evaluates the relation of the relative quantity of applied dose of PE and/ or IA and its achieved therapeutic effectiveness within the treated underlying neurological disorders. In a retrospective study, we evaluated data from PE and IA carried out 09/2009-06/2014 in neurological patients at the University-Hospital of Saarland, Germany. Apheresis dose was defined as the ratio of the extracorporeal treated plasma volume to the patient’s plasma volume. Effectiveness was assessed through disease-specific tests and scores by the attending neurologist(s); results were classified into response or no response. 1101 apheresis (PE:238, IA:863), in 153 hospital-stays were carried out, averaged, 7.0 treatments per patients, 82% responded, 18% not. Mean applied apheresis dose per treatment was 0.91 with mean doses of 1.16 for PE and 0.81 for IA. The totally applied mean dose per stay was 5.6 (PE:5.01, IA:5.81). No correlation was seen between apheresis dosing and treatment effectiveness (PE:R2 = 0.074, IA:R2 = 0.0023). PE and IA in therapy-refractory immune-mediated neurological disorders majorly achieved a measurable severity improvement – without correlation to the applied dose. Moreover, our data rather suggest, that effectiveness may be given with volumes below currently recommended volumes.

## Introduction

Apheresis is an established method of removing proteins and antibodies from the blood, here of interest plasmapheresis (PE) and immunoadsorption (IA), two methods with different targeted blood components. In PE, the patient’s plasma is withdrawn and replaced by fresh frozen plasma or by protein-contending solutions. In IA, the patient’s plasma is passed through an adsorber, eliminating the target components. PE and/ or IA are, at least in therapy-refractory cases^1^, the standard therapeutic option for immune-mediated neurological diseases, here chronic inflammatory demyelinating polyneuropathy (CIDP) Guillain-Barré syndrome (GBS), myasthenia gravis, or multiple sclerosis. Central pathogenic elements of these immune-mediated diseases are B-lymphocytes and antibodies as directed to structures of the nervous system and are involved in the process of neuroinflammation, leading to neurological symptoms e.g the ascending paralysis or sensory deficits. So far, recommendations for relative plasma volume per patient and treatment are few and inconsistent. In our study, we exactly ask the question of a direct proportional effectiveness of PE and IA regarding response/ no response correlated to the applied apheresis dose, meaning the relative quantity of treated plasma volume.

## Methods

### Study population

All patients with study relevant immune-mediated nlogeuroical disorders and treated in the Department of Neurology at the Saarland University Medical Centre (UKS) between Sept 2009 and June 2014, were per se eligible for this study. Thereof, the therapeutic option of apheresis was considered in case of therapy failure of steroids, immunoglobulins or in critically ill patients; the final indication for apheresis was provided by the Department of Neurology. Whereas, the choice of apheresis procedure (PE or IA) was determined interdisciplinary, based on the neurological disorder, individual risk factors and comorbidities of each patient. As this study evaluates clinical data purely retrospectively, written informed consent from all patients included was not obligatory, according to the vote of the local ethics committee (Landesärztekammer des Saarlandes; Ref. ID: 158/14). All clinical study data were obtained through revision of medical documentation and entered anonymously into our data bank.

### Apheresis treatment

All study incorporated apheresis treatments, PE and IA, were carried out by the Department of Nephrology UKS. For this purpose, the multifunctional device Octo Nova® was used. For IA, a Tryptophan-Immunadsorber (Immusorba® TR-350) from Diamed was used. In 4 cases the immunoadsorption device ADAsorb (Fresenius Medical Care) and the proteinA-adsorber Immunosorba® were used.

The actual study population was divided into three groups: patients treated by PE, treated by IA, or both – PE and IA – while reasons for change of procedure(s) were e.g., pre-existing treatment with ACE inhibitors, absolute contraindication for IA or allergic reactions on components of the donor plasma during PE.

### Apheresis dose

Apheresis dose was defined as the actual volume of plasma either exchanged during PE or treated during IA related to individual patients’ body plasma volumes. Actually exchanged or treated plasma volumes were taken from apheresis protocols; patient plasma volumes (PPV) were calculated according to Sprenger as following: Plasma volume [l] = 0.065 × body weight [kg] × (1-hematocrit). The single treatment relative apheresis dose is defined as the quotient of PE/ IA volume over patient plasma volume, while the cumulative administered apheresis dose per patient and hospital stay is the sum of all then applied treatment doses divided by the patient’s plasma volume. Both, effectiveness and clinical benefit were evaluated through disease-specific tests and scores by the attending neurologist. Effectiveness, classified into yes/ no response, was assessed for each hospitalisation. Both the administered single dose ratio per treatment as well as the sum dose ratio per hospital stay were correlated to the overall clinical benefit = yes/ no response.

### Method of anticoagulation for apheresis-treatment

Currently two options for anticoagulation are available: firstly, systemic anticoagulation with heparin (initial bolus and continuous application), or regional anticoagulation with citrate, infused to blood volume with a ratio of 1:32 and target concentration of post-filter ionized calcium 0.25–0.35 mmol/l as described by Calatzis [13].

### Complications during apheresis-treatment

Complications were classified into severe, moderate and mild complications; accordingly severe resulted partly in a complete abortion while other severe up to moderate led to discontinuation of single or pause of overall apheresis treatment with/ without potential drug therapy; mild complications often allowed to continue apheresis treatment, led seldomly to spontaneous stop, some were treated by a short-term mediation therapy.

### Assessment of the effectiveness of apheresis treatment

The effectiveness of PE and/ or IA was evaluated through determination of CIDP and GBS, MRC Sum Score^2^ and Overall Disability Sum Score (ODSS)^3^ before and after PE and/ or IA treatment. In responders, at least one of these scores was ameliorated afterwards. There against, no responders showed no improvement or even worsening of ODSS and MRC sum scale.

In more detail and/ or examples: In optic neuritis, visual acuity was examined by an ophthalmologist, here responders showed improvement of visual acuity after treatment. In cases of severe multiple sclerosis relapses, the expanded disability status scale (EDSS)^4^ was assessed, and responders’ EDSS score was at least 0.5 points lower after apheresis treatment. In myasthenia gravis, the besinger score^5^ was determined, and responders showed score improvement. In rare conditions (e.g. stiff person syndrome) without established scores yet available, the treating neurologists assessed potential benefits of apheresis to the patients.

### Statistical analysis

Continuous variables are expressed as mean ± SD (normally distributed variables) or as median and the range minimum to maximum (skewed variables). Categorical variables are presented as a percentage, unless otherwise stated. For comparison(s) between continuous variables the unpaired t-test was used. Categorical variables were compared by use of Chi-square test or Fisher exact test, respectively. Two-sided p-values <0.05 were considered as statistically significant. Data analysis was performed using IBM SPSS Statistics 19™ (IBM Corp., Armonk, N.Y., USA).

## Results

### Participants and descriptive data

During study period overall 3425 patients with immune-mediated neurological disorders within this study’s focus were treated: CIDP (n = 478), GBS (n = 41), myasthenia gravis (n = 305) and multiple sclerosis (n = 2601), in 102 patients (3.4%; 102/ 3425) PE or IA was carried out. One patient case was excluded due to insufficient available data. Overall, in 101 patients then 1101 procedures during 153 hospital stays (in-patients) were carried out; some patients were admitted to hospital more than once. Of 153 in-patients 31 were treated with PE, 102 with IA, and 20 with PE and IA. Basic characteristics of the final in-patient study population are presented in Table 1.

**Table 1:** baseline characteristics of 153 hospital stays.

	hospital stays
number	153
male	68% (104/153)
age (years)	56.1 (±17.4; 18.3–88.5)
BMI [kg/m^2^]	26.5 (±13.2; 18.5–48.8)
Treatment in ICU	13% (20/153)
SAPS II Score	26.5 ± 13.2
artificially ventilated	11%
Death during hospital stay	2.6% (4/153)

### Choice of anticoagulation

**Figure 1:**
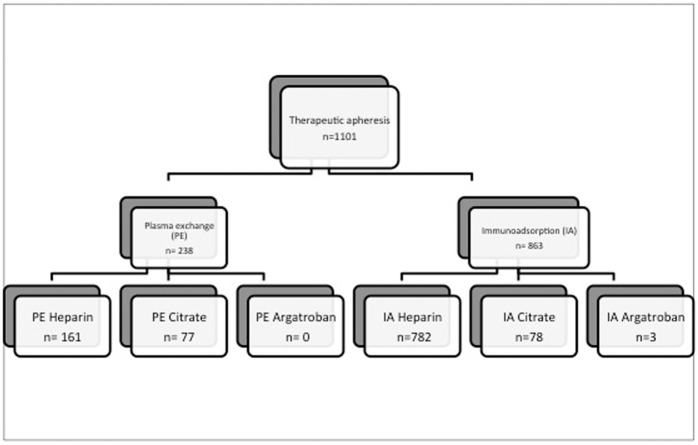
PE and IA and choice of anticoagulation.

Distribution of anticoagulation is shown in Fig. 1. (Fig. 1***:**** plasma exchange and immunoadsorption and choice of anticoagulation)* For anticoagulation mainly heparin was used. Mean applied dose of heparin was 5.965IE (1.000 – 17.000IE) with higher amounts in PE compared to IA (6835 vs. 5680IE). Overall, regional anticoagulation was used in 36/153 in-patients, in detail in 155/1101 therapeutic apheresis. In 16 in-patients both, heparin and citrate were applied. In one case of suspected but not confirmed HIT II argatroban was used for anticoagulation in 3 IA.

### Clinical benefit and effectiveness results

**Figure 2:**
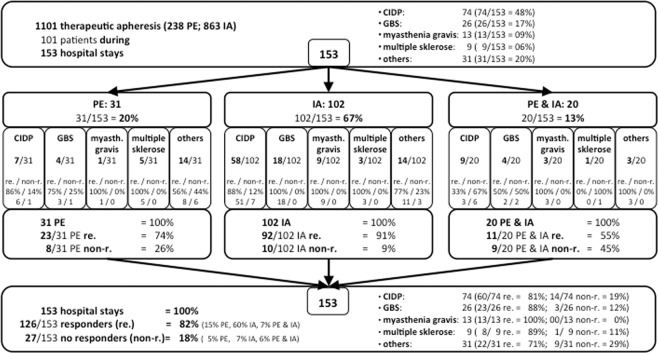
Clinical response to apheresis within 153 in-patients.

82% responded to apheresis during hospital compared to 18% non-responders as shown in Fig. 2.

### Apheresis dose

**Figure 3:**
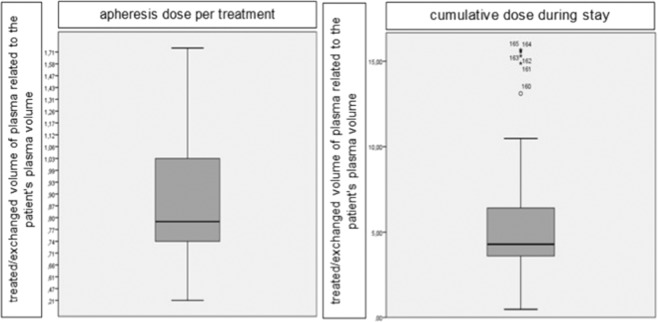
Mean apheresis dose per treatment and cumulative dose during stay.

Mean apheresis dose per treatment was 0.91 (0.21 to 1.72) times the patients’ plasma volumes. During one stay cumulative apheresis dose was 5.6 (0.47 to 64.8) times the PPV as shown in Fig. 3.

Mean apheresis dose of each PE (1.16; 0.5 to 1.7) was higher than of each IA (0.81; 0.2 to 1.6). In contrast, cumulative apheresis dose was lower for PE (5.0; 1.0 to 15.7) compared to IA (5.8; 0.5 to 64.7).

### Effectiveness and apheresis dose correlation results

In a nutshell, apheresis dose per treatment was not correlated to effectiveness. Mean apheresis doses per treatment in patients with clinical response or no clinical response were 0.89 and 0.97 times the patients’ plasma volume (coefficient of correlation R^2^ = 0.014). There was also no correlation between cumulative treatment dose per stay and effectiveness (coefficient of correlation R^2^ = 0.003). Mean apheresis doses per stay in patients with or without response were 5.9 and 5.4 times the patients’ plasma volume. Moreover, the effectiveness of applied apheresis doses was independent from chosen apheresis procedures (PE or IA or both).

### Complications

Overall, in 248 of 1101 treatments (22.5% - 248/1101 treatments) 270 complications occurred, in some cases 2 complications occurred simultaneously. Severe complications were rare in 3 of 270 (1.1%), moderate complications occurred in 42 of 270 (15.6%) and most complications were mild with 225 of 270 (83.3%). Interestingly, complications occurred two times more often in PE 37% (88/238 PE) compared to IA 18.1% (156/863 IA).

**Figure 4:**
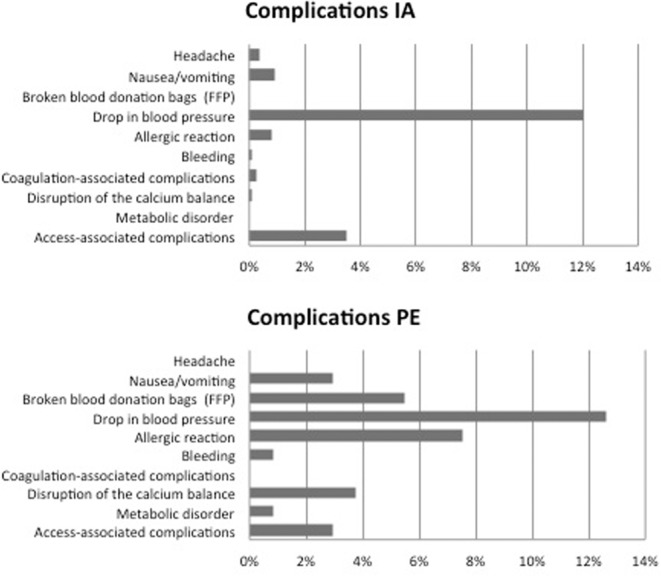
A/B: complications in PE an IA. (**A**) complications occurring for IA. (**B**) complications occurring for PE.

In a similar way, complications were more often under anticoagulation with citrate compared to under heparin (38.9% versus 19.5%). Occurrence of different complications is shown in Fig. 4A,B. (Fig. 4A,B*: complications in PE or IA–4 A: complications occurring for IA, 4B: complications occurring for PE)*.

## Discussion

Therapeutic plasma exchange (TPE) was described for the first time in 1914 as an extracorporeal blood purification technique^6^. Today, TPE is an established method for the treatment of immune-mediated neurological disorders^1^. However, there are only few and inconsistent recommendations with respect to the relative quantity of plasma volume to be treated. This study shows that the applied apheresis dose ratio does not seem to influence like-wise proportionally the actual effectiveness of therapeutic apheresis in immune-mediated neurological disorders.

Historical development of TPE supports this finding as therapeutic apheresis has already been applied in the sixties with total volumes of 500-700 ml, independently from patients’ plasma volumes^7^. In the early 1980s, new techniques of plasma separation then started to allow treatments with higher total volumes as such^8^.

In principle, studies show that antibody removal is subject to a saturation curve with a relative optimum at a 1-fold plasma volume exchange, meaning removing about 50% of the antibodies^9^. But, plasma exchange against human albumin containing solutions is restricted in its relative maximum due to the loss of proteins, complement and coagulation factors limiting the TPE frequency and lowering the achievable/ possible reduction of antibodies^9^. However, in the 1980s higher exchange volumes were described with 1.5-2 fold plasma volume^8^. In contrast, currently therapeutic apheresis is recommended 5-7 times within 10-14 days, with replacement volumes between 1 and 1.5 times the patients’ plasma volumes^1,10^. However, as far as our literature research revelled current relative volume recommendations have not yet been evaluated in prospective studies of considerable size on apheresis dose and its proportional effectiveness, even less on immune-mediated neurological disorders as of interest here. The few studies with a mentionable number of patients (68 to 419) and treatments (388 to 1945)^11–14^ rather report about quite heterogeneous underlying diseases and varying doses. These studies do not allow to draw clear conclusions for relative dose recommendations regarding effectiveness per se and/ or proportionality.

Now, our current study is not only based on one of the largest numbers of evaluated TPEs and patients but additionally purely assesses a restricted category of underlying diseases, solely immune-mediated neurological disorders.

Following the ASFA recommendations, an average of 7.1 treatments was carried out within our study cohort, while the mean apheresis dose (0.91 times PPV) was lower than suggested. Though, the positive apheresis response rate of 82% within these otherwise standard therapy-refractory cases was comparable to other studies^12,13^. Moreover, there was no correlation between the applied relative volume per treatment and/or the relative cumulative dose during one hospital stay and the proportional effectiveness of apheresis as such – instead, the study cohort could be divided into responders and non-responders per se.

A few small studies indicate similarly, that effectiveness of apheresis may be also given with volumes below recommendation: PE with treated plasma volume of 0.6 times the patients’ plasma volume in optic neuromyelitis^15^, showed comparable effectiveness to the usually applied doses (1-1.2 times PPV)^16^. Another study evaluated the impact of the number (not volume!) of treatments in GBS on effectiveness, showing firstly an improvement and then a saturation curve: Increasing treatments from 2 to 4 in mild and moderate cases resulted in higher effectiveness, but an increase in the severe cases up to more than 4 treatments did not achieve any additional benefit^17^. A third case showed as well effectiveness of apheresis in myasthenic crisis despite treatment volumes below recommendation^1,18^.

Overall, in literature treatment of immune-mediated neurological disease is predominantly described with PE. But, removing pathological antibodies from the patient’s blood is also possible with IA. Here, due to saturation of the binding sites, the plasma volume being effectively treated with most plasma-adsorber, is limited to volumes of 2 to 2.5 litres^19^. Higher plasma treatment volumes can be reached e.g. with proteinA-adsorber allowing treatments of high volumes (1.5 to 2.5 times PPV). However, removal of proteins limits the frequency and total volume of application of IA: a therapy volume of 2.5 litres, reduces antibodies by 65–75%, fibrinogen by 75% and albumin by 25%^20^. The reduction of fibrinogen leads to pronounced hypofibrinaemia in 20% of IA treatments^21^. An increasing number of studies report about successful application of IA in standard therapy-refractory cases of immune-mediated neurological disorders. However, effectiveness of PE and IA was evaluated only in a few studies so far showing comparable effectiveness^18^ in different neurological disorders with clinical benefit in 71% in steroid-refractory cases^22^ to up to 100% in patients with neuromyelitis optica^23^. Although our evaluation is retrospective, positive response rate to IA is comparable with studies reporting about PE.

## Limitations

Although our study is limited due to the retrospective design a correlation between apheresis dose ratio and clinical benefit should have been visible with respect to the high number of patients and treatments. The treated plasma volume is varying due to technical reasons, complications or clinical evolution.

## Conclusions

Therapeutic apheresis is a promising therapeutic tool in therapy-refractory cases of immune-mediated neurological disorders. PE and IA show comparable clinical benefit in our study, independently from the relatively applied apheresis dose. Our results, such as others, seem to show increasing evidence that the needed apheresis dose for effectiveness in treatment responders is below the historically grown dose expectations and actual recommendations. Therefore, our results are promising and should be confirmed in prospective studies.
